# Seasonal Variation of Lead in Fish Pond Waters of High Hunting Activity Area and Relation to Metals and Ions

**DOI:** 10.1007/s11270-014-2217-1

**Published:** 2014-11-18

**Authors:** Łukasz J. Binkowski, Bartłomiej Rzonca

**Affiliations:** 1 Institute of Biology, Pedagogical University of Cracow, Podbrzezie 3, 31-054 Kraków, Poland; 2 Institute of Geography and Spatial Management, Jagiellonian University, Gronostajowa 7, 30-387 Kraków, Poland

**Keywords:** Lead, Pollution, Anions, Hunting activity, Monitoring, Water

## Abstract

Anthropogenic activities such as industry, agriculture, and daily life are related to metal pollution of the environment. Places known of the highest impact are fishponds where intensive fish farming is believed to input a significant amount of various elements to water. Additionally, many studies suspect wetland hunting activity of water lead pollution. The present paper aims to check if hunting is a significant source of lead (Pb) in water as well as to study the temporal trends of numerous parameters (pH, SEC, Cd, Cu, Zn, Ca, Mg, Na, K, NH4+, HCO_3_^−^, SO_4_^2−^, Cl^−^, NO_3_^−^, F^−^) in ponds (*n* = 48) and inflow (*n* = 24) waters near Zator in southern Poland, Europe. Most concentrations were measured with ion chromatography and electrothermal atomic absorption spectrometry. Lead concentrations in pond waters were low and found not to be linked with hunting activity, as well as they did not differ from the ones found in the inflow water. Moreover, it could be stated that activities led on ponds did not enrich rivers in the studied ions and elements.

## Introduction

The flow waters and inland water bodies serve human economy and, as no other, are put under economic and production pressure. A particular type of inland water bodies are water ponds. These basins are characterized by strongly altered chemical composition of water, being the result of deliberate man’s actions (e.g., related to fish farming) and other factors. Water pollution, on the other hand, makes water organisms vulnerable to poisoning. Research concerning water birds shows that environment pollution has a great impact on their organisms and pollutants’ bioconcentration (Kertész et al. 2006; Babińska et al. 2008; Binkowski and Meissner 2013). Apart from pollution, being the result of intensive fish culture economy, the chemical condition of waters is also influenced by agriculture and industry, as well as angling and hunting taking place on water basins’ banks. While the influence of industry is being widely researched, the one of angling and hunting remains to a large extent unknown, even though it may be the source of additional, fairly significant threats. For instance, lead weights and pellets used by anglers and hunters may constitute a significant source of water pollution (Pain 1990; Scheuhammer and Norris 1995). Some of the pellets shot during hunting, having missed the target, land on the ground (Pain 1990, 1991a). One ammunition cartridge used in Poland for water birds (ducks, geese and coots) hunting contains an average of 34 g of lead (caliber 12, no. 4). In shooting of a single bird, a few of the shots always miss the target, which means that in the sites of intensive huntings yearly, up to even a few hundreds of kilograms of lead are accumulated (Hughes 2002). It is suspected that such pellets, lying on the water basin’s bed, may undergo the process of pulping in water, causing its pollution (Wilk et al. 2010). Dissolution of metals, lead pellets included, depends—to a large extent—on the chemical-physical qualities of the water, e.g., its reaction.

Animals living in the wetland areas, places rich in deposits, are exposed to numerous elements and compounds, not only lead. Additionally, it turns out that it is not only cadmium and lead but also other metals, such as copper and zinc, which may constitute a threat for ecosystems and living organisms (Nordberg et al. 2007). They are commonly used in man’s economy, and their concentration in the environment is substantial. The problem, however, is the establishment of concrete concentrations in the environment for which these elements—being ultra elements, in particular concentrations indispensable to live—are a threat for organisms.

The research studies on water birds in the Zator water ponds area, in numerous cases, showed the increased concentrations of cadmium (strong nephrotoxin) and lead (Binkowski et al. 2013a, b; Binkowski and Sawicka-Kapusta 2015a, b). The concentrations were big enough to cause histological lesions in tissues of the studied birds (Binkowski et al. 2013b). Further research on lead only showed the increased concentrations in nearly ¼ of the studied birds. The conclusions of the research on birds from the ponds of Zator generate the question about the mechanism and source of the metal pollution. In the case of lead, one of the most frequently suspected mechanisms (apart from the erroneous swallowing of pellets as gastrolites—Pain 1991b) is the mechanism of lead pellets dissolution in pond waters and contamination of birds’ organisms via polluted water and food (e.g., water plants) consumption. In the case of cadmium, the source of the metal is not explicitly defined, yet most probably, it is related to the bottom residues.

In the present research, our aim was to verify the hypothesis, whether the pond water contamination causes the metal contamination of the water birds’ meat. In order to investigate the issue, we systematically monitored the chemical composition of the water ponds in Zator, where increased cadmium and lead concentrations were noted in mallards and coots (Binkowski and Sawicka-Kapusta 2015a, b). Monitoring the dynamic of numerous parameters through the whole season was the target of the study. The subject of our research was not only the content of the selected heavy metals in water but also other metals, such as copper, zinc and other physicochemical water features, which might influence the mobility and assimilation of metals. We also assessed the coincidence of the particular lead concentrations and the start of the water bird hunting season.

## Materials and Methods

**Fig. 1:**
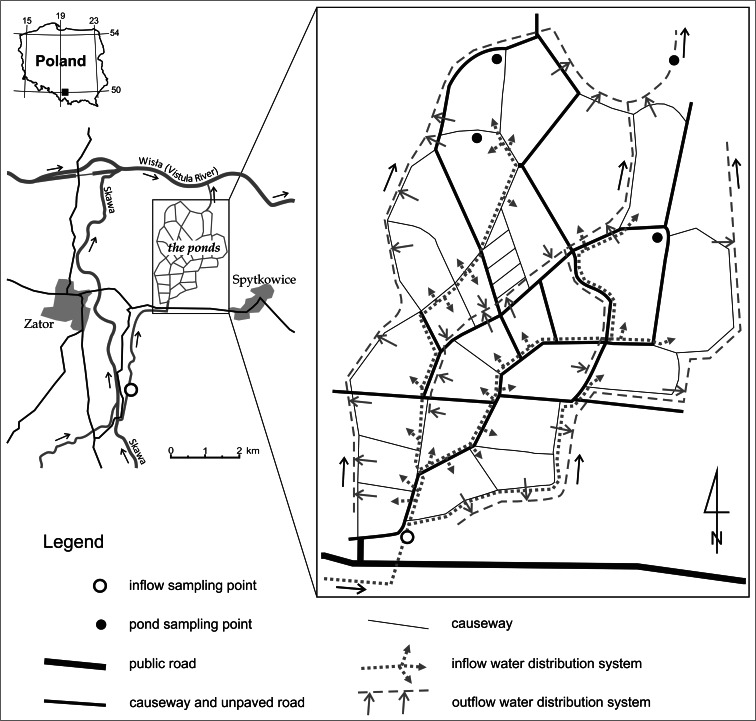
Area of carried research—fishponds around Zator (c.a. 40 km from Cracow) in southern Poland

The research was conducted in the water ponds of the “Spytkowice” Rybacki Zakład Doświadczalny (Fishing Experimental Unit), a fish farm in Zator (belonging to Instytut Rybactwa Śródlądowego—the Inland Fisheries Institute—in Olsztyn). The research area is located on the Kraków—Oświęcim main Polish country route around 40 km from Kraków in southern Poland (Fig. 1). The farm is a vast complex of ponds with the total water surface of 4.2 km^2^ (diked area of nearly 4.9 km^2^). In the unit, many fish species are bred, mainly carp, pike, catfish, and amur. The ponds’ depth reaches 3 m maximum, 2 m in average. In the pond area, there are few trees; therefore, nearly the whole water surface is well exposed to sunrays. Apart from agriculture and fishing, another frequent activity in the area is water birds and marsh birds hunting, with particular intensity in between August and November (the hunting season). The intensity of this type of hunting is one of the highest in Poland.

The research consisted in the systematic observation of the waters’ chemical composition. It had been conducted for 12 months from March 2006 to February 2007. Six constant sites of sample collection were chosen—two from the Skawa river (inflow water to a pond complex) and four from ponds (Fig. 1). The Skawa River is a medium-sized river in the Carpathian Mountains, Poland. It is a right tributry of the Vistula River. It springs out at the height of around 700 m above sea level below the Spytkowicka Pass (also Bory Orawskie Pass), situated on the continental divide. The river has a complex river regime with spring snowmelt, and summer rain surges. Even though the Skawa drains the low and medium-sized mountains, its regime shows typically mountainous features with high and sudden summer rain surges. The river flows into the Vistula near Zator, in the outskirts of the Carpathian Mountains at the height of 217 m above sea level. At the mouth of the river, its average flow (SSQ) equals 17.2 m^3^/s. In the river basin area, one can notice relatively high anthropopressure on the quality of groundwater related to unsatisfactorily well-organized sewage management (The Atlas of Water-level Gauge Posts Atlas posterunków wodowskazowych 1996; Chełmicki et al. 1999).

At the beginning of each month, water samples were collected into plastic bottles of 0.5 L volume and cooled to the temperature of 4 °C. The samples were transported to the laboratory within 24 h. All the necessary conditions concerning the collection, conservation, and keeping of the samples required by the laboratory were carefully fulfilled. Once, during a year-long study (in June), in the sites of samples collection, the local pH, and water conductance (Elmetron pH/Conductivity meter CPC-401) were measured in order to determine the impact of transport on the researched parameters—it turned out insignificant.

In the laboratory, water pH and conductance (SEC) were measured (both with Elmetron CX-742). The SEC changes were treated as a representative picture of total dissolved solids (TDS). Ion chromatograph (Dionex ICS-2000) with analytical columns AS18 and CS16 (4 and 5 mm, consecutively; both by Dionex) was used to determine the contents of the major ions (Ca^2+^, Mg^2+^, Na^+^, K^+^, NH_4_^+^, HCO_3_^−^, SO_4_^2−^, Cl^−^, NO_3_^−^, F^−^). In turn, the contents of the selected heavy metals (Cd, Cu, Pb, Zn) in water were analyzed by means of the graphite furnace atomic absorption spectrometer (PerkinElmer AAnalyst 800). For the heavy metals and ion concentration analyses, the practical limits of detection and determination were considered and calculated (Fleming et al. 1997) (Table 1).

**Table 1:** Limits of detection and quantification of IC and ET-AAS method used in analyses

	Limit of detection	Limit of quantification
Cd [μg/L]	0.055	0.118
Cu [μg/L]	1.074	1.750
Pb [μg/L]	0.346	0.614
Zn [μg/L]	1.078	1.563
Ca [mg/L]	0.005	0.015
Mg [mg/L]	0.005	0.015
Na [mg/L]	0.01	0.03
K [mg/L]	0.005	0.015
SO_4_^2−^ [mg/L]	0.01	0.03
Cl^−^ [mg/L]	0.0025	0.0075
HCO_3_^−^ [mg/L]	0.025	0.075
F^−^ [mg/L]	0.001	0.003
NH_4_^+^ [mg/L]	0.005	0.015
NO_3_^−^ [mg/L]	0.0025	0.0075

The results of the Shapiro Wilk and Levene tests revealed that the data do not meet the assumptions of the parametric tests. Therefore, for the collected data, the nonparametrical analysis of variance for repeated measurements (Friedman ANOVA) was also conducted in order to verify the presence of any statistically significant differences in water parameters of particular months. A separate analysis (with the Mann–Whitney *U* test) allowed to compare the differences of the parameters between water types (inflow and pond waters). The variability of the studied parameters was presented by means of a median with minimal and maximal values. All the calculations and analyses were conducted with the use of the following software: Microsoft Excel 2010 and StatSoft STATISTICA 10.

## Results

**Fig. 2:**
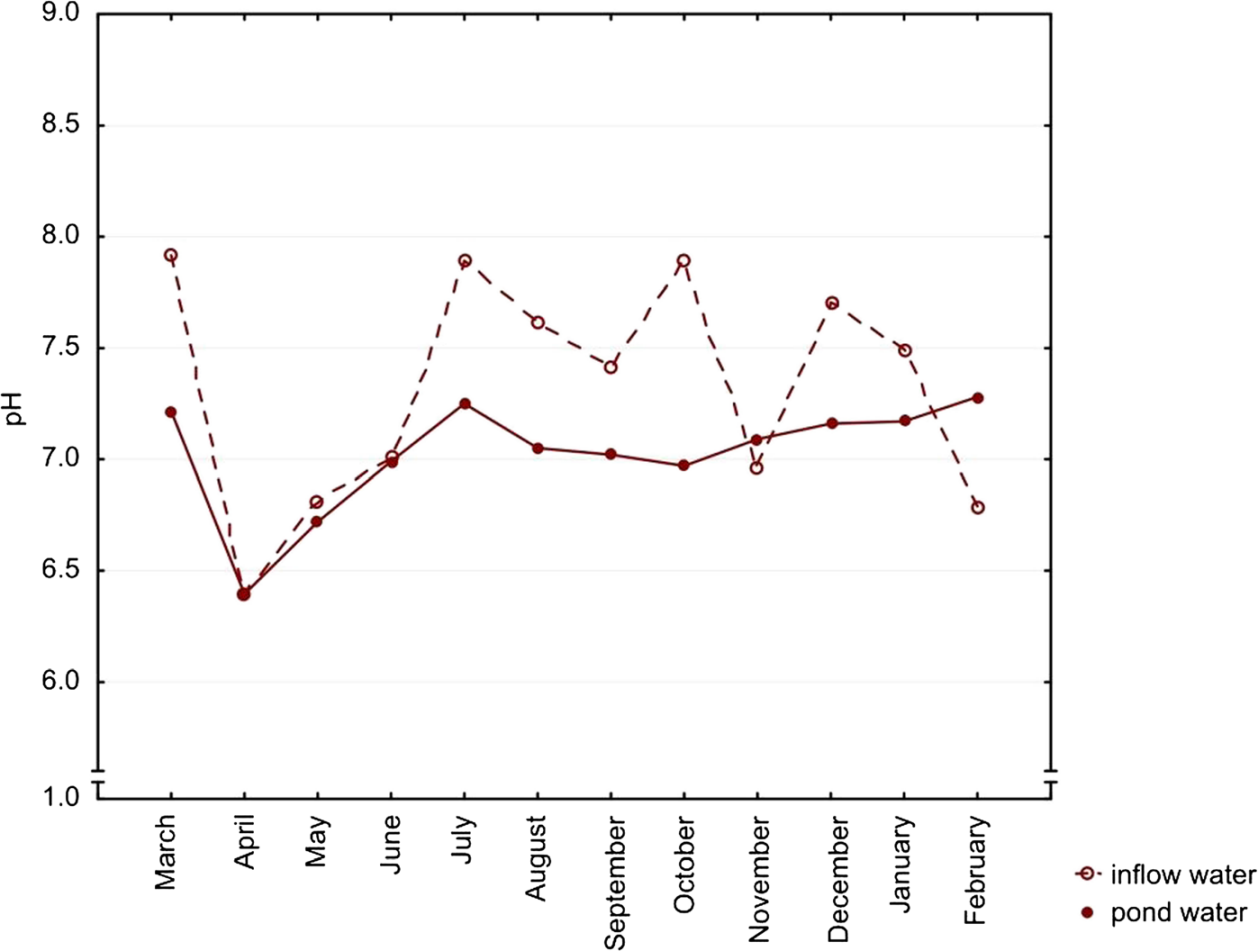
Seasonal variation of median pH values. Differences between types of water, *p* = 0.0239. Seasonal variation in inflow water (*p* = 0.0504) and in pond water (*p* = 0.6935)

**Fig. 3:**
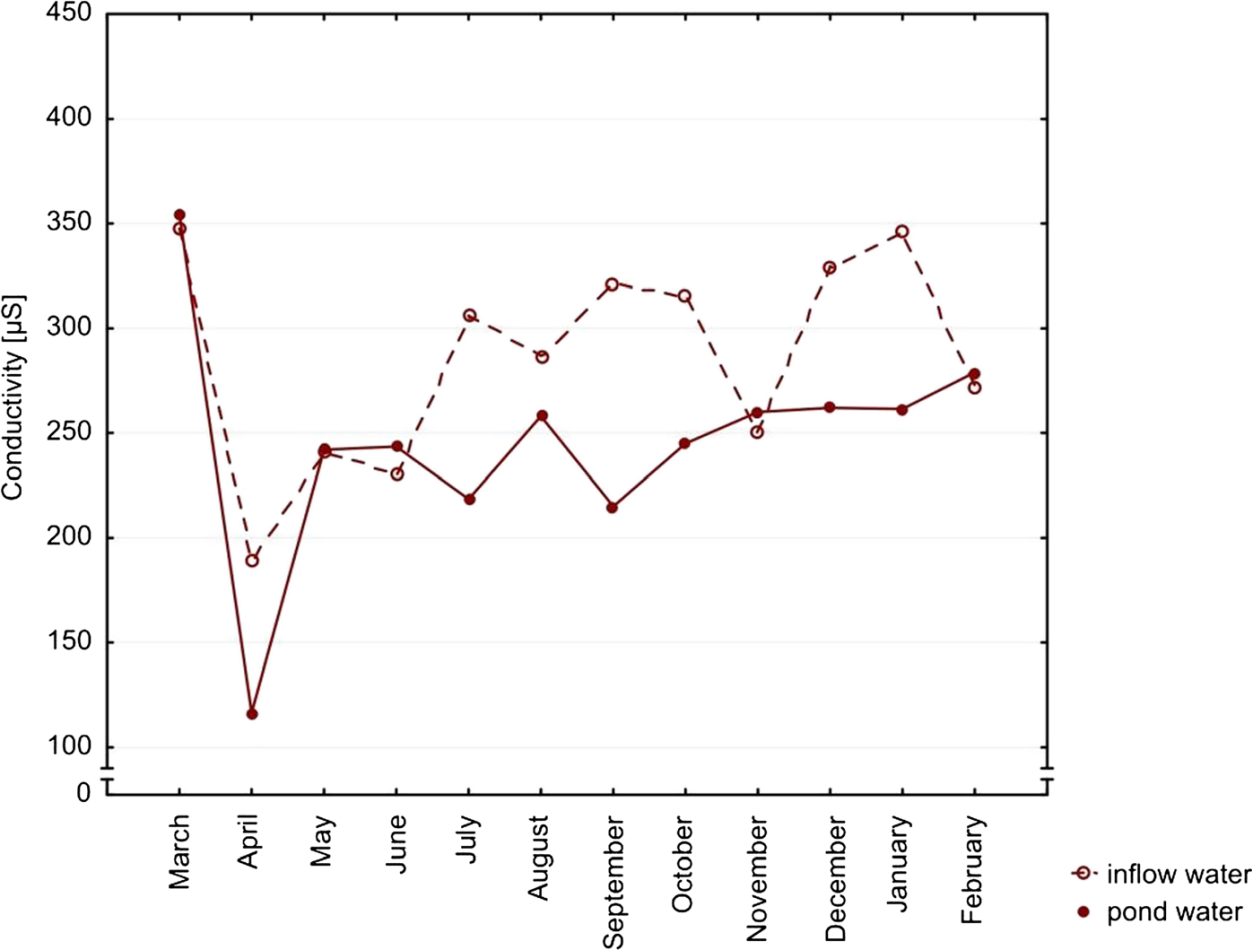
Seasonal variation of median conductivity values. Differences between types of water, *p* = 0.0189. Seasonal variations in inflow water (*p* = 0.0443) and in pond water (*p* = 0.1948)

The air temperature on sampling days varied between months. The mean value was 13.6 °C. The minimum value −6 °C was noted in February, the highest one 30 °C occurred in July. Main physicochemical characteristics of the studied waters as pH and specific electric conductivity differed significantly as regards the source of water (Table 2). Lower median pH value was noted in the pond waters (7.09) than in the inflow samples (7.42). We observed similar tendency in the case of conductivity 256.00 and 303.00 μS/cm, respectively. Additionally, in the inflow water pH, as well as SEC, varied significantly according to the seasonality factor (pH variation was at the significance level). Both parameters in both types of water had the lowest medians in April and the highest in March (Figs. 2 and 3).

**Table 2:** Studied parameters of water with statistical comparison according to the type of water and seasonality (12 consecutive months)

	Inflow water (*n* = 24)					Water type *p* ^a^	Pond water (*n* = 48)				
	Median	Min	Max	Seasonality^b^	Season range^c^		Median	Min	Max	Seasonality^b^	Season range^c^
pH	7.42	6.35	8.34	0.0504	See Fig. 2	*0.0239*	7.09	6.14	9.88	0.6935	See Fig. 2
Conductivity [μS]	303.00	171.40	359.00	*0.0443*	See Fig. 3	*0.0189*	256.00	23.60	399.00	0.1948	See Fig. 3
Cd [μg/L]	0.00	0.00	0.15	*0.0396*	See Fig. 4	0.8428	0.00	0.00	7.49	0.3816	See Fig. 4
Cu [μg/L]	2.28	0.00	8.87	0.4798	See Fig. 5	0.8435	1.98	0.00	16.16	0.0725	See Fig. 5
Pb [μg/L]	0.74	0.00	7.57	0.0765	See Fig. 6	0.9433	0.74	0.00	2.48	0.0776	See Fig. 6
Zn [μg/L]	1.84	0.76	14.86	0.2642	See Fig. 7	0.5230	1.86	0.83	43.00	0.1623	See Fig. 7
Ca [mg/L]	44.02	26.68	54.77	0.0571	↓ April ↑ January	*0.0224*	37.92	3.19	58.83	0.0929	↓ September ↑ March
Mg [mg/L]	6.22	3.25	9.07	*0.0334*	↓ April ↑ October	0.3262	5.76	0.31	8.88	0.1034	↓ April ↑ October
Na [mg/L]	10.79	3.31	13.36	*0.0289*	↓ April ↑ September	0.1271	8.84	0.65	13.67	0.1079	↓ April ↑ March
K [mg/L]	2.98	1.47	4.62	*0.0403*	↓ April ↑ February	0.3562	2.77	0.28	5.11	0.0597	↓ April ↑ March
SO_4_^2−^ [mg/L]	26.88	16.44	39.60	0.0991	↓ June ↑ March	0.0668	24.22	1.71	58.33	0.1274	↓ July ↑ March
Cl^−^ [mg/L]	12.90	4.99	18.62	*0.0341*	↓ April ↑ March	0.0930	10.86	1.06	18.35	*0.0403*	↓ April ↑ March
HCO_3_^−^ [mg/L]	131.51	64.04	166.35	*0.0413*	↓ April ↑ September	*0.0342*	117.84	10.95	175.20	0.3753	↓ September ↑ March
F^−^ [mg/L]	0.07	0.01	0.13	*0.0358*	↓ January ↑ October	0.2003	0.07	0.01	0.15	0.0991	↓ June ↑ August
NH_4_^+^ [mg/L]	0.08	0.03	0.47	0.1056	↓ May ↑ March	0.4063	0.14	0.04	0.65	0.1158	↓ December ↑ March
NO_3_^−^ [mg/L]	7.17	1.66	12.70	0.0303	↓ October ↑ March	*≤0.0001*	2.09	0.62	9.43	0.3812	↓ August ↑ March

Values set in italics indicate statistically significant differences

^a^ *p* value of Mann–Whitney *U* test

^b^ *p* value of ANOVA Friedman test

^c^↓— the lowest median; ↑—the highest median

Bicarbonate anion and calcium cation dominated in both types of water (Table 2). We also noted relatively high concentrations of sodium cation, as well as sulfate and chloride anions. Among all the studied ions, only calcium, bicarbonate, and nitrate concentrations differed statistically between waters from different origins. In the inflow waters, cadmium, magnesium, sodium, and potassium, as well as chloride, bicarbonate, fluoride, and nitrate concentrations, varied significantly through the 1-year study. On the contrary, only chloride anion concentrations showed this trend in the pond waters (Table 2).

**Fig. 4:**
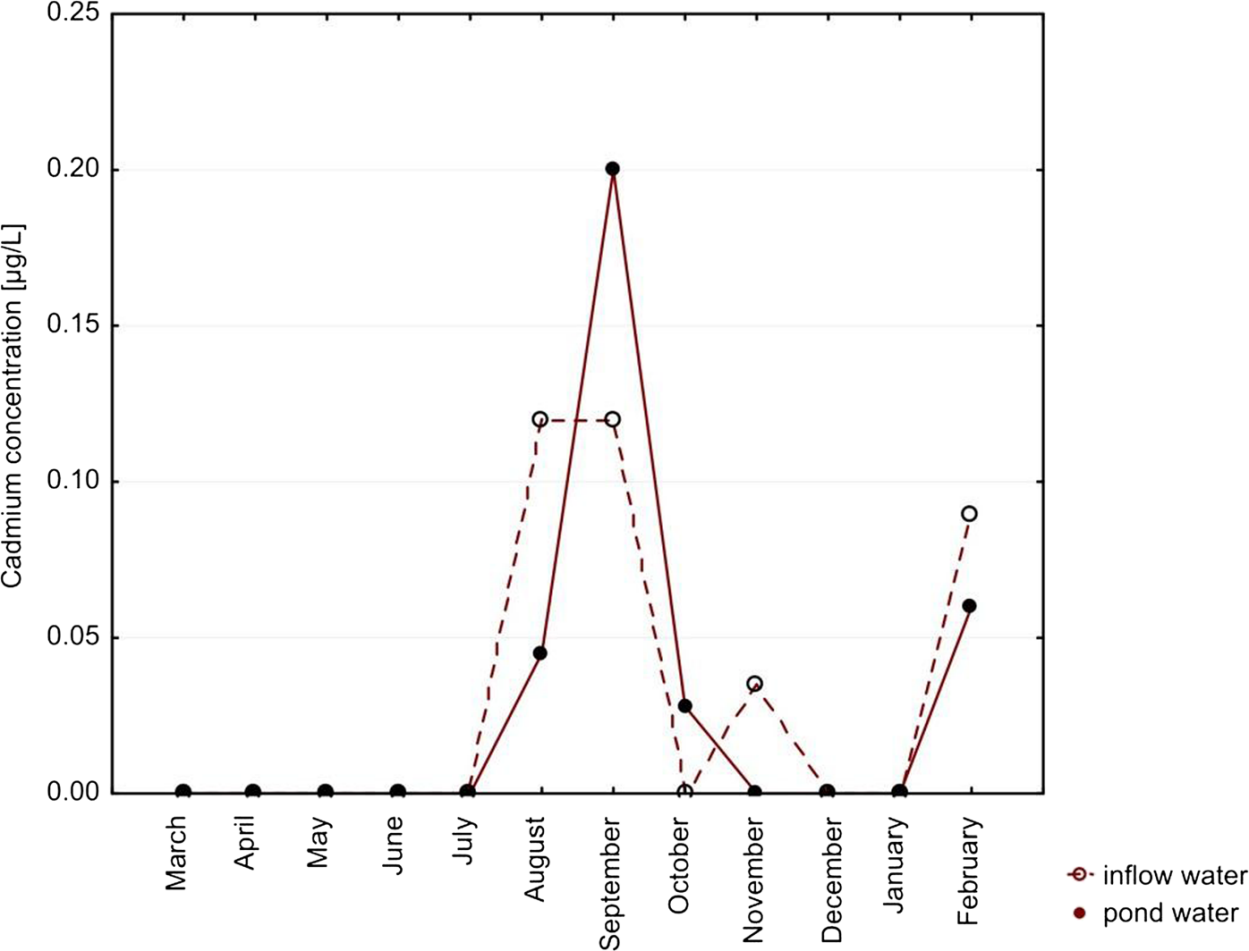
Seasonal variation of median cadmium concentrations. Differences between types of water, *p* = 0.8428. Seasonal variation in inflow water (*p* = 0.0396) and in pond water (*p* = 0.3816)

**Fig. 5:**
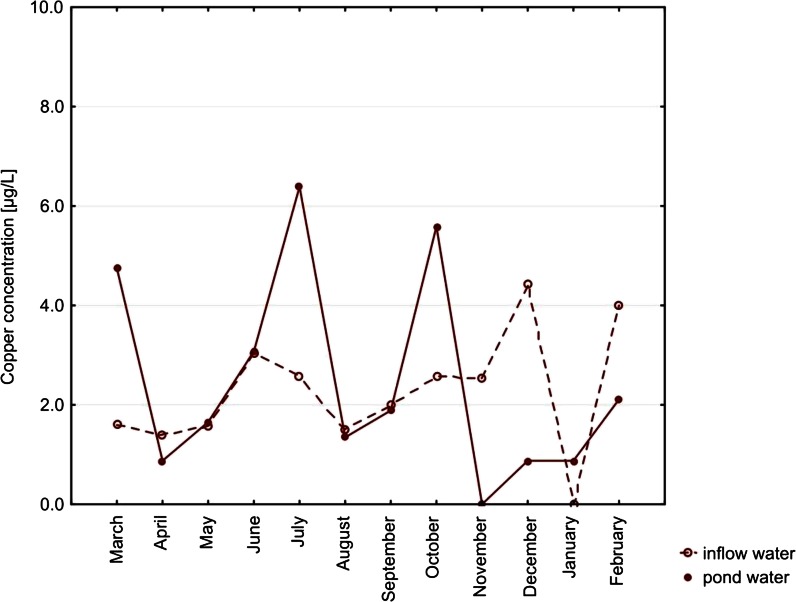
Seasonal variation of median copper concentrations. Differences between types of water, *p* = 0.8435. Seasonal variation in inflow water (*p* = 0.4798) and in pond water (*p* = 0.0725)

**Fig. 6:**
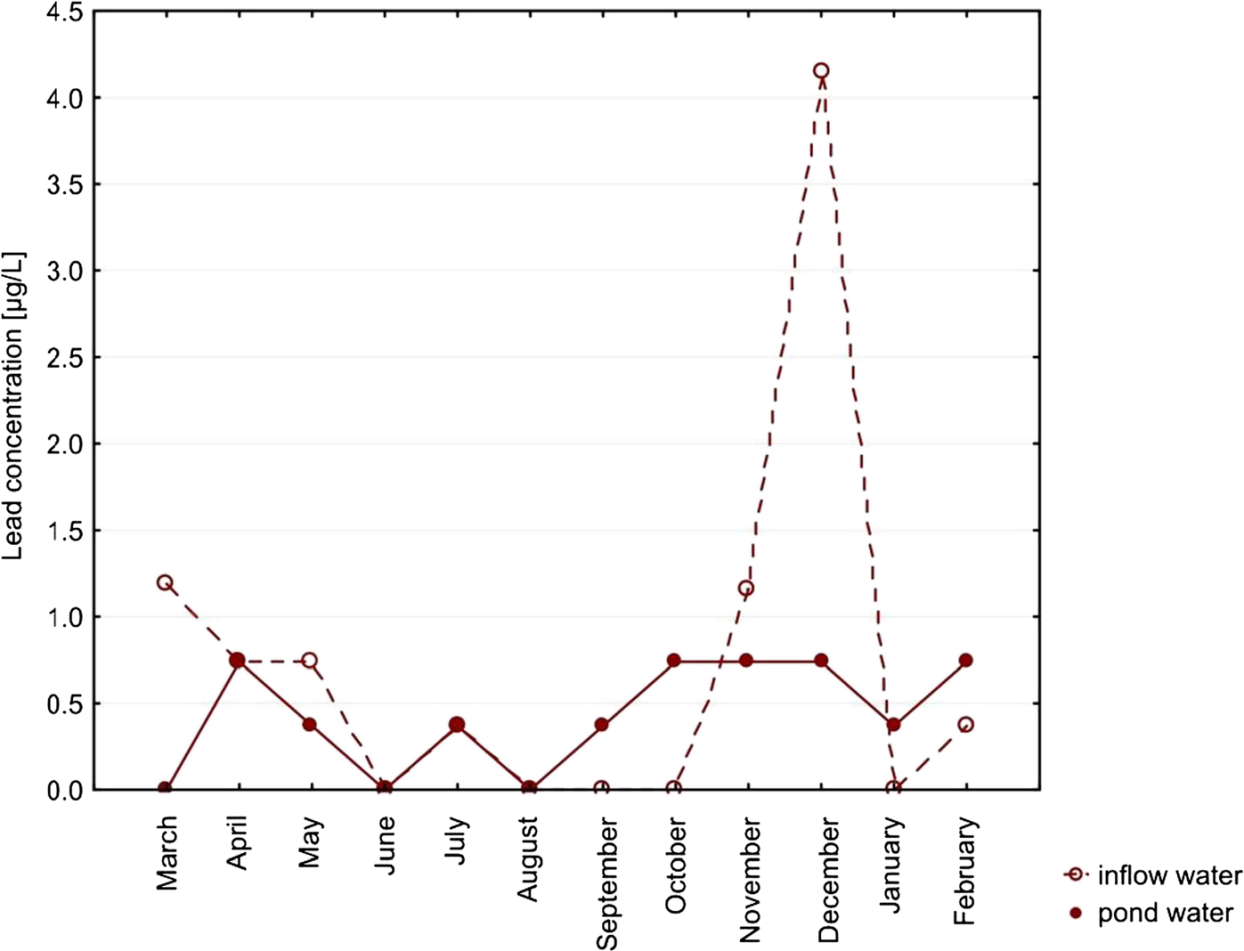
Seasonal variation of median lead concentrations. Differences between types of water, *p* = 0.9433. Seasonal variation in inflow water (*p* = 0.0765) and in pond water (*p* = 0.0776)

**Fig. 7:**
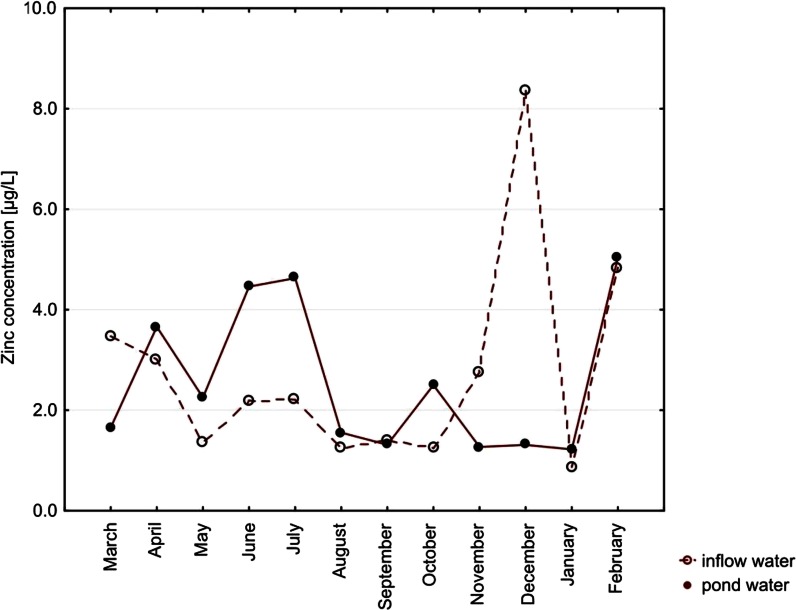
Seasonal variation of median zinc concentrations. Differences between types of water, *p* = 0.5230. Seasonal variation in inflow water (*p* = 0.2642) and in pond water (*p* = 0.1623)

Among metals, the highest concentrations were noted for copper (up to 16.16 μg/L) and zinc (up to 43 μg/L) in pond waters. Median concentrations of cadmium in both types of water, with no month distinction, were lower than the limit of detection. Lead concentrations did not exceed 7.57 μg/L (noted for inflow water). Concentrations of all the metals did not differ significantly between the types of water (Table 2). Only in the case of cadmium, the concentration in the inflow water’s seasonality was a significant factor (*p* = 0.0396; Figs. 4, 5, 6, and 7).

Lead concentrations were negatively correlated with six parameters: the calcium, magnesium, sodium, potassium cations, hydrogen carbonate anion concentrations, and conductivity. Zinc concentrations were negatively correlated with the pH value, sodium, ammonium, and chloride ions concentrations. Concentrations of copper and cadmium were related with air temperature on the sampling day. Moreover, cadmium concentrations were also positively correlated with ammonium and fluoride ion contents (Table 3). Among metals themselves, statistically significant relationship occurred only between concentrations of copper and zinc (*R* = 0.5929).

**Table 3:** Statistically significant correlations between concentrations of metals and water and environmental parameters

Metal concentration	Water parameter	*R* Spearman
Cd	Air temperature	0.3604
	NH_4_^+^	0.2603
	F−	0.3493
Cu	Air temperature	0.2761
Pb	Conductivity	−0.3460
	Ca	−0.2494
	Mg	−0.2861
	Na	−0.3041
	K	−0.2473
	HCO_3_^−^	−0.3526
Zn	pH	−0.2848
	Na	−0.2957
	NH_4_^+^	−0.3018
	Cl^−^	−0.2466

## Discussion

We found that, with the exception for cadmium, the trace element concentrations in both studied waters did not differ due to the seasonality factor. What is more, the differences in the mentioned concentrations between waters from inflows and ponds were not statistically significant. Lead and zinc concentrations were negatively correlated with numerous studied ions, whereas cadmium and copper concentrations were positively correlated with air temperature and cadmium—also with two ion concentrations.

## Representativeness of the Studied Population of Samples

The regular taking sample program made it possible to monitor the studied waters’ seasonal chemical composition changes. As far as the hydrometeorological situation is concerned, the year of the carried research turned out to be a relatively representative year. There were neither greater water freshets (floods) nor particularly deep low waters (hydrological drought). Therefore, the population of the researched samples meets the temporal representativeness requirements.

The samples used in the study were taken in six constant sites. Two of them allowed for monitoring of the chemical composition of river waters feeding ponds, whereas the pond area was sampled in the other four sites. Researchers consider such a number of sites and their distribution sufficient to notice accidental mistakes, e.g., a single sample pollution, as well as to separate strictly local phenomena in the sense of a single sampling point from the general ones, i.e., typical, e.g., for the ponds or the feeding river. Simultaneously, the analysis of the spatial differentiation of the water chemical composition was possible for ponds.

## General Characteristic and Variation Between Water Types

TDS and pH were slightly higher in the inflow waters, but generally in both of the sample groups, the reaction was neutral and the conductivity was low. Both types of water were dominated by calcium and bicarbonate which are considered common in the literature for surface and ground waters (Macioszczyk and Dobrzański 2002). Concentrations of these ions showed a significant variation between water sources—mostly they were lower in the pond waters (Table 2). The literature data (Hermanowicz et al. 1999) reported that the average content of magnesium in the Polish surface waters is about four times lower than that of calcium. Our studies confirm that the amount of calcium is much higher, however, with an average multiple around 7. According to the same paper (Hermanowicz et al. 1999), the sodium content in Polish waters is in average four times higher than the potassium one. Our results confirmed this tendency (Table 2).

Higher maximum values noted for Cd, Cu, and Zn occurred in pond waters, whereas Pb content was lower (Table 2). Generally, the quality of water from ponds, as regards metals content, let us even consider it as drinking water (Kabata-Pendias and Pendias 1999). In comparison to the water from Polish lakes, the noted concentrations of all the metals were significantly lower (Szymanowska et al. 1999). Various studies from Poland showed that cadmium occurs mainly at very low levels, which is consistent with our results (Kabata-Pendias and Pendias 1999; Ostrowska 2005; Table 2). Concentrations of copper, lead, and zinc in the studied waters were visibly lower than average values noted for Poland (Kabata-Pendias and Pendias 1999; Ostrowska 2005) where lead concentrations reached even 60 μg/L, which is many times more than in the studied waters (Table 2). Probably, due to low acidity of water, lead does not migrate from the pellets to water and is embedded in deposits (Mateo et al. 1997; 2000). Similar deposition is suspected also for other metals (Dałkowski et al. 2005). The mentioned observations let us conclude that waters in the studied area were not polluted with metals including lead. The study also revealed that both types of studied waters did not differ in the aspect of metal concentrations (Table 2). We can state then that various types of activities undertaken in the fishpond areas, such as fish farming and hunting, do not input a significant amount of metals to pond water. Moreover, ponds—places were intensive fish farming and hunting activity takes place—do not enrich rivers (where they outflow the water) in studied ions and elements. Except for the daily pollution (sewages input), probably the main source of metal in the inflow and pond waters was connected to the geological weathering of rock and soil exposed to water (Ochieng et al. 2006).

## Seasonal Differences

In the pond waters, seasonal differences were significant only for chloride, whereas in the inflow waters, numerous elements (including cadmium) and ions varied across months (Table 2). Lead, which was suspected to come from the hunting activity (Wilk et al. 2010), did not change its concentrations according to the hunting season which starts annually on 15th August and lasts in the studied area technically till the end of November.

In the inflow water, pH values were slightly lower (statistically insignificant but very close to the significance, *p* = 0.0504) in March and April. It may be linked to the increasing temperature and dissolving residual layer of ice when snowmelt waters recharge rivers. This, in turn, stimulated the increase of the activity of ions which is equivalent to the lowering of the pH of the water. Significantly, the lowest SEC in the inflow waters was observed in spring, which is probably connected with the smallest input of organic matter to water (Jokiel and Tomalski 2005) and dilution by the meltwater. According to the literature, we suspected higher concentrations of most ions and elements in summer because of water evaporation (Singh et al. 2007). However, we did not observe such a trend among the studied compounds, so probably, the inflow of water from the Skawa River constantly compensated for the water loss in ponds.

Generally, the study revealed that the pond waters’ chemical composition was significantly more stable in comparison to that of the inflow waters which may be explained by the big pond capacity. Thus, the chemical character of the pond waters can be weakly changed by inflowing water, which is characterized by strong seasonal variability typical for the river waters. The visible increase in lead concentrations in inflow water in December was unique during the whole year of study, hard to explain, and not correspondent to observations found in ponds. The parameters of pond waters are more determined by the ongoing processes in ponds rather than the chemical characteristics of small inflow. However, a significant variation in numerous parameters in temporary ponds was noted by Arle (2002). This discrepancy can be linked with a completely different water budget in temporary ponds, which is enclosed only to precipitation and evaporation.

## Relationships

We did not find any significant correlations among metal concentrations so probably their sources in the studied waters were different (Table 3). Among them, we can mainly speculate about the agriculture, geochemistry background, sewages, and air pollution. Positive relationships with water parameters occurred only in the case of cadmium and copper where the air temperature seemed to play a significant role in the solubility increase. Zinc and lead were correlated with a few parameters only in the negative direction. Lead concentrations were linked with calcium and magnesium contents. There is some evidence in the literature that lead solubility in water depends on water hardness (Sorensen 1991). Both abovementioned ions are strictly connected with the hardness, as well as with TDS which is usually higher in hard water. Generally, we suspected to find, in the studied waters, the negative correlations between metal concentrations and pH which may be explained by the chemistry of water. However, we did not find such relationships at least with the studied pH values range, except for zinc (the lower pH, the higher Zn concentrations). Probably, relatively low concentrations of other metals did not let us observe the mentioned correlation.

## Conclusions

In comparison to the inflow waters, ponds were very stable water bodies. Both types of waters in the studied area were not polluted with metals. Additionally, lead concentrations did not correlate with the start of hunting season so hunting activity is not a source of this metal in the studied water. All the metal concentrations did not vary between the water sources. We conclude then that the activities carried out in the fishpond areas do not enrich water in metals in a different way than agricultural activities nearby the inflows. Additionally, we can state that fishpond water is not a significant source of metals for water birds foraging in the studied area.
